# Rethinking ALS: Current understanding and emerging therapeutic strategies

**DOI:** 10.3934/Neuroscience.2025021

**Published:** 2025-09-25

**Authors:** Arosh S. Perera Molligoda Arachchige

**Affiliations:** Faculty of Medicine, Humanitas University, Via Rita Levi Montalcini 4, 20072 Pieve Emanuele, Lombardy, Italy

**Keywords:** amyotrophic lateral sclerosis, motor neuron disease, neurodegeneration, Riluzole, RNA metabolism

## Abstract

Amyotrophic lateral sclerosis (ALS) is a fatal neurodegenerative disorder characterized by progressive degeneration of the upper and lower motor neurons, which leads to muscle atrophy, spasticity, and ultimately respiratory failure. The etiology of ALS remains unclear, though a combination of genetic and environmental factors is suspected. Advances in understanding ALS pathophysiology, including the role of RNA metabolism, mitochondrial dysfunction, and glutamate toxicity, have paved the way for new research directions. While Riluzole offers limited survival benefits, there is no cure, and treatment remains mostly supportive. This article summarizes the current understanding of ALS in terms of its pathophysiology, epidemiology, risk factors, clinical presentation, and treatment strategies.

## 1. Introduction

Amyotrophic Lateral Sclerosis (ALS), also known as Lou Gehrig's disease, is a progressive neurological condition that primarily affects motor neurons [1]. The hallmark of ALS is the simultaneous degeneration of the upper and lower motor neurons, which control voluntary muscle movements [2],[3]. This degeneration leads to a gradual loss of muscle function, and culminates in paralysis and respiratory failure [4]. Despite extensive research, the precise cause of ALS remains elusive. However, understanding its pathophysiology could lead to improved management and supportive care [5]. This review provides an updated synthesis of therapeutic strategies as of 2024–2025, thereby highlighting recently approved agents, key negative trials, and imaging biomarkers that may soon change the associated clinical practice. By integrating very recent data on drug withdrawals and emerging gene-targeted therapies, this article offers an up-to-date resource for non-neurology care teams and specialists alike, distinguishing it from earlier ALS overviews.

## 2. Pathophysiology

ALS primarily affects both the upper motor neurons (UMN) in the corticospinal tract and the lower motor neurons (LMN) in the anterior horn of the spinal cord [6]. The term “amyotrophy” refers to the atrophy of muscle fibers caused by denervation due to the degeneration of the anterior horn cells [7]. “Lateral sclerosis” refers to changes seen in the lateral columns of the spinal cord, where UMNs degenerate, thus leading to gliosis (i.e., scarring by astrocytes) [8].

ALS progression is believed to involve several interconnected mechanisms that contribute to the degeneration of motor neurons. One such mechanism is an aberrant RNA metabolism, which involves the dysfunction of TAR DNA-binding Protein 43 (TDP-43), a protein crucial for mRNA regulation. This disruption can impair gene expression and axonal transport, further destabilizing neuronal function [9]. Another significant factor is mitochondrial dysfunction, where mutations in genes such as the Coiled-coil-helix-coiled-coil-helix domain containing 10 (CHCHD10) compromise the respiratory chain, which results in oxidative stress and eventual neuronal death [10].

Excitotoxicity also plays a key role, which is driven by excess glutamate that overstimulates the motor neurons and leads to their degeneration. Mutations such as those within superoxide dismutase 1 (SOD1) are associated with altered glutamate transport and receptor function, thus amplifying this effect [11]. Additionally, glial cell dysfunction contributes to the progression of ALS. In particular, the RNA/RNA-binding proteins in astrocytes and microglia, which typically provide critical support to neurons, become dysfunctional and exacerbate neuronal death, further accelerating disease progression. These overlapping mechanisms collectively create a complex pathology that underlies ALS [12],[13].

## 3. Epidemiology

ALS is a relatively common motor neuron disorder, with an incidence of 1–3 cases per 100,000 and a prevalence of 1–9 cases per 100,000 globally [14]. ALS is uncommon in individuals under 40 years old, with its prevalence rising sharply with increasing age. The average age of onset ranges from 58 to 63 years for sporadic ALS and between 40 and 60 years for familial ALS. The highest incidence is observed in individuals aged 70 to 79 years [15]. Men are slightly more likely to develop ALS than women, with a lifetime risk of 1 in 350 for men and 1 in 420 for women [16].

Geographic differences in ALS incidence have been observed, with higher rates in populations such as the Chamorro people of Guam and the Auyu and Jakai people of New Guinea [17],[18]. Finland also shows a notably higher incidence, nearly double the global average [19].

## 4. Etiology and risk factors

The etiology of ALS remains largely unknown. However, both genetic and environmental factors contribute to its development [20]–[22]. About 5%–10% of ALS cases are familial, with genetic mutations such as SOD1, TAR DNA Binding Protein (TARDBP), RNA-binding protein fused in sarcoma (FUS), and chromosome 9 open reading frame 72 (C9orf72) being commonly implicated [23]–[25]. Mutations in these genes account for most familial ALS and illuminate key molecular pathways. For example, C9orf72 repeat expansions disrupt RNA metabolism and cause toxic dipeptide repeat proteins, while SOD1 mutations lead to oxidative stress and protein aggregation. TARDBP and FUS mutations impair RNA splicing and transport. The penetrance varies, and some carriers remain asymptomatic until late adulthood, thus underscoring complex gene–environment interactions [23]–[25].

Sporadic ALS accounts for most cases, where no clear familial link is present [26],[27]. Smoking is the only well-established environmental risk factor, with an odds ratio (OR) of 1.38, thus indicating a moderately increased risk [28]. Military service and participation in sports, such as soccer, have been suggested as potential risk factors, possibly due to an exposure to toxins or repeated trauma [29],[30]. In some populations, exposure to environmental toxins, such as the cycas micronesica plant, has been proposed as a contributing factor [31]. Moreover, occupational exposure to electromagnetic fields, as encountered by pilots and electricians, has been suggested as a possible contributor, although the evidence remains inconclusive [32].

## 5. Clinical presentation

The clinical presentation of ALS is varied, but typically begins with subtle signs of muscle weakness, usually in the distal limbs [33]. Early symptoms include difficulties with fine motor tasks, such as buttoning clothes, tripping due to foot drop, and muscle cramps. Fasciculations, or muscle twitching, are common but often overlooked by patients until they become more pronounced. As the disease progresses, muscle atrophy, spasticity, and hyperreflexia become evident [34].

In some cases, ALS may present in atypical forms, such as progressive muscular atrophy that primarily affects LMNs, which leads to asymmetrical muscle weakness, and primary lateral sclerosis, a slower form of ALS with predominant UMN involvement, which is characterized by spasticity and brisk reflexes [35],[36].

Bulbar involvement affecting the face, throat, and tongue can lead to dysphagia and dysarthria. Bulbar-onset ALS often causes aspiration due to impaired airway protection well before classic respiratory muscle weakness. In contrast, distal limb onset usually progresses to thoracic involvement with eventual respiratory failure [37].

ALS is a relentlessly progressive disease, with a median survival time of 3–5 years from the onset of symptoms. Younger patients and those with limb-onset ALS tend to have better outcomes, with about 5% of patients living longer than 10 years. Death is usually due to respiratory failure or complications related to immobility, such as aspiration pneumonia.

Venous thromboembolism (VTE) is an often-overlooked complication, with immobility and muscle wasting contributing to increased risks [38].

## 6. Diagnosis

The diagnosis of ALS is primarily clinical, based on the presence of both upper and lower motor neuron signs.

Diagnosing ALS is a complex process that requires the exclusion of other disorders with similar clinical features. The El Escorial criteria are commonly used, and requires evidence of upper and lower motor neuron degeneration, the progressive spread of symptoms across regions, and an absence of alternative explanations for the clinical presentation, which is confirmed through electromyography (EMG), motor conduction studies, and neuroimaging [39],[40]. Providing a definitive diagnosis and conveying it to the patient can be challenging, which is why referring the patient to a highly specialized clinical center with an experienced multidisciplinary team for a second opinion is suggested [41].

The diagnostic criteria for ALS rely on three core principles:

1. Presence of functional impairment in a specific body region;

2. Clinical evidence of both UMN and LMN involvement in one or more segmental anatomical areas; and

3. Progression of functional decline.

A diagnosis remains uncertain without fulfilling all three criteria and requires re-evaluation [39].

EMG plays a crucial role in the diagnostic workup for ALS. EMG, combined with nerve conduction studies, serves to identify conditions that mimic ALS and to demonstrate the loss of motor units, which is a hallmark of ALS pathology. The diagnostic framework is based on the revised El Escorial criteria, which divide the motor regions into bulbar, cervical, thoracic, and lumbosacral. UMN involvement typically presents as spastic paresis, subtle reflex changes, and reduced myotatic reflex thresholds, often in visibly atrophied muscles [39],[42].

EMG identifies LMN lesions by revealing reduced motor response amplitudes, slowed conduction speeds, and the presence of fibrillations and sharp waves. Additionally, it can help exclude ALS-mimicking disorders such as myasthenia, myositis, motor neuropathy, and conduction block. The Awaji-Shima criteria highlight fasciculations as significant diagnostic markers when observed alongside chronic neurogenic changes in motor unit potentials (MUPs) [43],[44].

**Figure 1.**
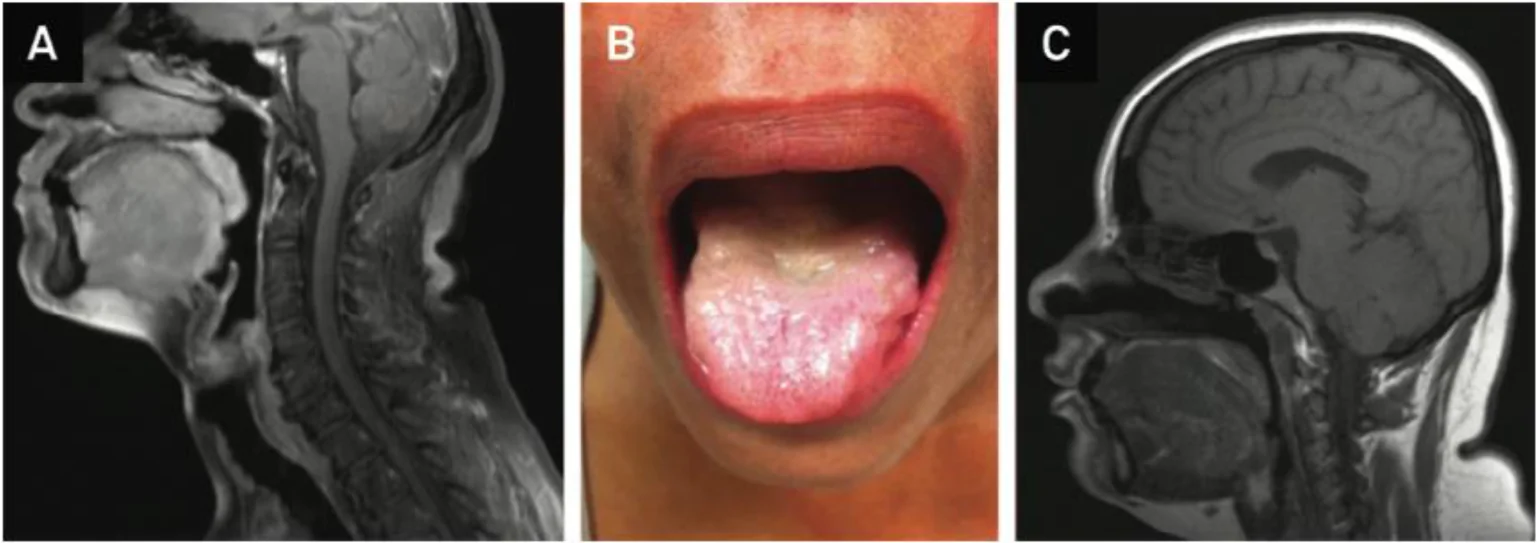
Sagittal T1-weighted brain MRI discloses abnormal diffuse hyperintensity of the tongue musculature (A), correspondent to severe atrophic tongue (B). Normal tongue MRI feature is showed [45].

Meanwhile, magnetic resonance imaging (MRI) offers complementary structural insights. Although an MRI of the neuroaxis in ALS may be normal, several characteristics—but non-specific—findings have been described. T2 hyperintensity in the corticospinal tracts is a classical feature, and is seen earliest in the internal capsule where the fibers are most concentrated. As the disease progresses, hyperintensity and volume loss may extend along the entire tract from the motor cortex to the spinal cord. However, this finding is only present in approximately 30% of cases, with a sensitivity below 40% and specificity under 70%, which necessitates cautious interpretations. On T1-weighted imaging, the “bright tongue sign”—hyperintensity of the tongue—may be observed in patients with bulbar involvement [45], see Figure 1.

**Figure 2.**
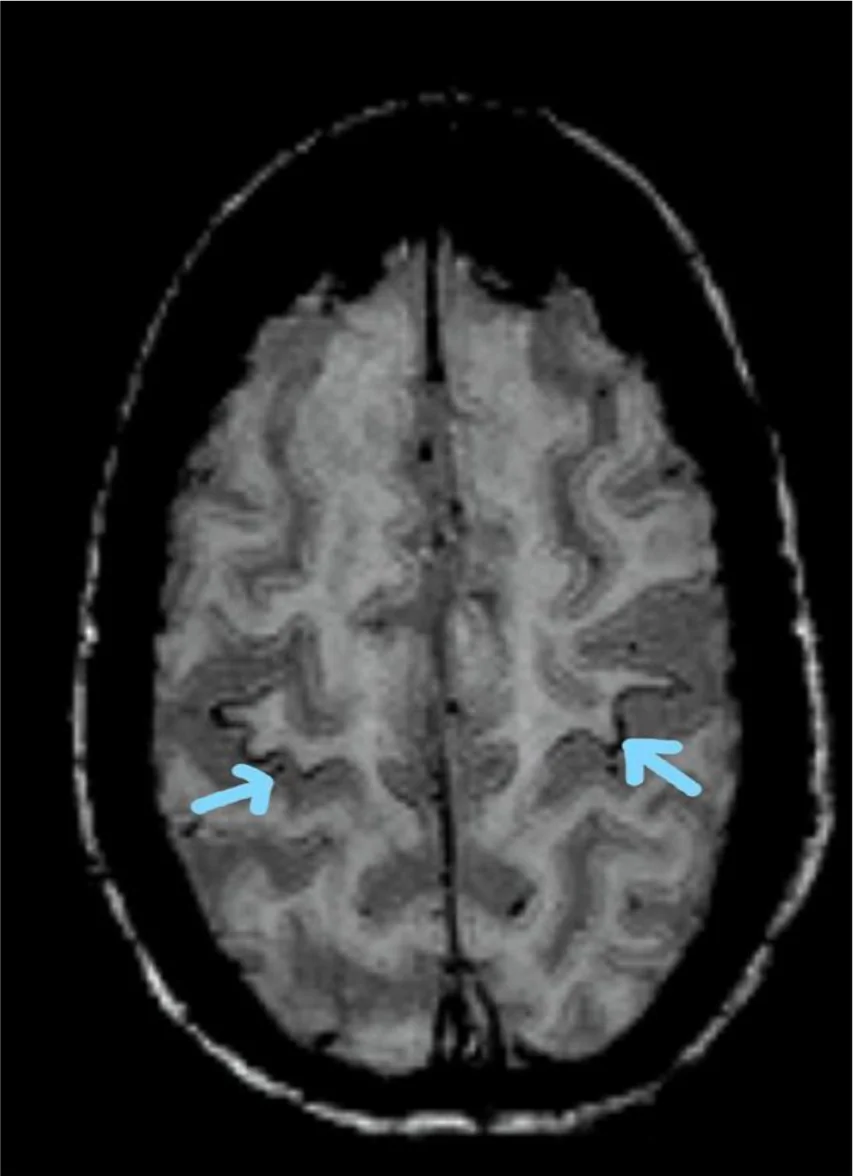
Bilateral hypointense signals in the precentral gyri (blue arrows) on a SWI sequence, demonstrating the motor band sign in a patient with confirmed primary lateral sclerosis (PLS) [46].

Gradient echo sequences (GRE) and susceptibility weighted imaging (SWI) sequences may reveal bilateral hypointensity in the precentral gyri, known as the “motor band sign”, which can be more specific than corticospinal tract hyperintensity, as it may be seen in patients lacking the latter but not vice versa [46], see Figure 2.

MR spectroscopy adds a metabolic context, typically demonstrating decreased N-acetylaspartate (NAA) and glutamate, along with increased choline and myo-inositol. A spinal MRI is generally less helpful, and is primarily used to exclude mimics such as cervical myelopathy, although subtle features such as a decreased cross-sectional area of the cervical spinal cord—especially at C4–C7—may occasionally be present.

The revised El Escorial criteria and Awaji-Shima criteria collectively guide ALS diagnoses, with the latter emphasizing early detection through fasciculations and electrophysiological findings [47],[48].

## 7. Treatment and management

Given the incurability of the disease, conventional treatments for ALS are centered on maximizing the quality of life using disease-modifying drugs.

In addition to managing and slowing the course of ALS symptoms, novel treatments use innovative techniques such as genetic targeting, neuroprotection, and anti-inflammatory tactics to improve the patients' circumstances and consequently their quality of life. Instead of only concentrating on symptom treatment, these therapies aim to directly address the underlying mechanisms of the disease, thereby presenting the possibility of improved neuroprotection, decreased inflammation, and even disease change [49].

### 7.1. FDA approved drugs

As of 2023, the FDA has approved seven drugs to treat ALS and its symptoms: Qalsody, Relyvrio, Radicava, Rilutek, Tiglutik, Exservan, and Nuedexta. Additionally, various potential treatments are undergoing clinical trials to evaluate their safety and efficacy before seeking FDA approval.

### 7.2. Qalsody (Tofersen)

Biogen's Qalsody, also known as tofersen or BIIB067, received FDA approval in April 2023 to treat a rare form of ALS caused by mutations in the SOD1 gene, which affect approximately 2% of ALS patients. This drug works by targeting the SOD1 mRNA, which reduces the production of SOD1 proteins, thereby slowing the decline in muscle function. Intrathecally administered Tofersen managed to reduce the concentrations of SOD1 in the cerebrospinal fluid (CSF) and of neurofilament light chains in plasma, which is a biomarker associated with nerve injury and neurodegeneration [50].

### 7.3. Relyvrio (AMX0035)

Developed by Amylyx Pharmaceuticals and approved by the FDA in 2022, Relyvrio (AMX0035) combines sodium phenylbutyrate and taurursodiol. Being one of the most promising therapies, the association of sodium phenylbutyrate-taurursodiol (PB-TURSO) has been designed to protect neurons from oxidative stress and mitochondrial dysfunction. These drugs have been tested in a randomized, double-blind, placebo-controlled CENTAUR trial and have demonstrated an average of 7 months increase in long-term survival benefit from the early initiation of PB-TURSO treatment in participants with ALS [51]. However, in 2024, the manufacturer voluntarily withdrew Relyvrio from the market after the phase 3 PHOENIX trial failed to show a significant benefit over the placebo.

### 7.4. Radicava (Edaravone)

Radicava (edaravone), from Mitsubishi Tanabe Pharma America, became the first new ALS-specific treatment approved by the FDA in 2017 after a 22-year gap. Its oral formulation was subsequently approved in 2022 [52].

### 7.5. Riluzole

Covis Pharma's Rilutek (riluzole) was the first FDA-approved ALS treatment in 1995. It works by inhibiting glutamate release and can slow disease progression and extend survival by a few months, thus making it an effective treatment for ALS. Riluzole, the generic version of Rilutek, has been available since 2003. Riluzole is often used alongside other therapies, such as physical therapy and respiratory care, to manage ALS symptoms [53].

Additionally, ITF Pharma's Tiglutik, a thickened liquid form of riluzole, was approved by the FDA in 2018. This formulation avoids complications that can arise from crushing tablets [54]. Subsequently, Exservan, an oral film formulation of riluzole developed by Mitsubishi Tanabe Pharma America, received FDA approval in 2019. Designed for patients with severe swallowing difficulties, it dissolves on the tongue, thus eliminating the need to swallow pills or liquids [55].

### 7.6. Nuedexta

Nuedexta, approved by the FDA in 2011 and manufactured by Otsuka America Pharmaceutical, is prescribed for the pseudobulbar affect (PBA). PBA causes frequent and uncontrollable episodes of crying or laughing that are disproportionate to actual emotions [56]. As a combination of dextromethorphan HBr and quinidine sulfate, Nuedexta has proven effective in treating this condition, which is often associated with ALS and other neurological disorders. Moreover, early observational studies suggest off-label benefits for swallowing and speech in patients with bulbar weakness.

### 7.7. Supportive measures

Supportive interventions, such as non-invasive ventilation (NIV) and percutaneous endoscopic gastrostomy (PEG), can improve the quality of life and prolong survival in ALS patients [57].

Excessive salivation can be distressing and increase aspiration risk. First-line therapy includes anticholinergic agents (e.g., glycopyrrolate) or botulinum toxin injections. For refractory cases, salivary gland ligation or low-dose radiation therapy may provide durable relief [58],[59].

Trials are ongoing for other potential treatments, including drugs that target mitochondrial function and anti-apoptotic agents.

### 7.8. Drugs under investigation

Currently, some phase 2 and 3 studies have been completed and report discordant conclusions on their use, despite the necessity of more studies to truly understand their potential.

The combination of ciprofloxacin/celecoxib (PrimeC) is under investigation, having recently passed safety and tolerability tests, for its possible use [60].

This drug has been designed to target multiple mechanisms involved in ALS, including inflammation, iron accumulation, and impaired RNA regulation. It combines ciprofloxacin and celecoxib, which are two drugs that synergistically act to address motor neuron degeneration and reduce neuroinflammation. In Phase 2 trials, PrimeC showed a significant slowing of disease progression as measured by the ALS Functional Rating Scale (ALSFRS-R) and is now in Phase 3 clinical evaluation [60].

Regarding symptoms, Mexiletine is a drug that showed its potential in the control of ALS related muscle cramps. This Class 1B antiarrhythmic (weak sodium channel blocker) is largely used to suppress ventricular arrhythmias, but recently proved effective in the treatment of myotonic dystrophy to alleviate muscle pain and severe myotonia [61]. Thus, its application for the management of ALS symptoms has been investigated, and it was proven to be a well-tolerated and effective medication to control muscle cramps [62].

Pridopidine, a sigma-1 receptor agonist originally studied in Huntington's disease, is also under investigation for ALS due to its potential neuroprotective properties [63].

Another promising therapy involves Neurotrophic factors-secreting mesenchymal stromal cells (MSC-NTF cells): this is a novel cell-therapeutic approach aimed at effectively delivering neurotrophic factors to slow neurodegeneration directly to the site of damage in ALS patients [64] (Table 1).

Despite significant advancements in understanding the physiological and pathological mechanisms of ALS, many of the drugs developed have failed to meet their primary endpoints in clinical trials. However, these trials have provided valuable insights, including providing key biomarkers and new therapeutic targets. These discoveries enhance our understanding of the disease and provide a foundation for the development of more effective treatments in the future. This ongoing research offers hope for new strategies to combat ALS based on these critical learnings [65].

### 7.9. Surgical management

In treating ALS, surgical interventions are not therapeutic, and are generally aimed at managing symptoms and improving the quality of life rather than halting disease progression. Several studies have indicated that surgery can potentially accelerate the progression of the disease, specifically for spinal surgery. From the anesthetic point of view, the goal is to choose a technique that least interferes with the disease pattern but still provides good operating conditions and adequate analgesia. It presents its unique challenges, as ALS patients have a high risk of respiratory depression, and many have suggested that regional anesthesia techniques can be successfully employed to avoid these complications, though a study by Onders et al. suggested that general anesthesia can be safely performed during laparoscopic surgery in patients undergoing the installment of a diaphragmatic pacing system (DPS) [66]. For respiratory complications, diaphragm pacing was thought to provide temporary support by stimulating the diaphragm. Though previously approved by the US FDA, studies showed that patients with a DPS had a shorter survival than patients with non-invasive ventilation (NIV) alone. As procedure-related complications were also common, the review by Dorst et al. concluded that the installment of a DPS should not be considered as a therapeutic option for ALS [67]. NIV remains the standard of care despite its low utilization rate [68]. Tracheostomy is considered as second option, considered if NIV fails, thus enabling long-term ventilatory support. However, there are fears of “locked-in syndrome”, and of the high burden placed on caregivers. Tracheostomy has significant quality-of-life implications that should be discussed with the patients.

The placement of gastrostomy tubes (such as percutaneous endoscopic gastrostomy (PEG) or radiologically inserted gastrostomy (RIG)) has been found safe in ALS patients with minimal forced vital capacity (FVC) and advanced ALS though the American Academy of Neurological Societies and the European Federation of Neurological Societies guidelines recommend PEG placement before respiratory insufficiency (FVC < 50%) [69]–[71]; it becomes recommended for patients with severe dysphagia to maintain nutritional intake and prevent aspiration. Salivary gland ligation or radiation therapy can be performed for patients with sialorrhea after the first line of treatments such as botulinum toxin and acetylcholinergic agents fail [69]–[71].

Orthopaedic interventions, including tendon releases and spinal fusions, can relieve joint contractures and spinal instabilities, thus helping to enhance comfort and facilitate posture. It is important to note that unnecessary spine surgery should be avoided for ALS patients who might have potentially been misdiagnosed with a spine condition instead of ALS. Overall, surgical interventions in ALS are largely palliative, thereby focusing on symptom relief and comfort while balancing the significant ethical and quality-of-life considerations inherent in treating a progressively debilitating disease [72].

**Table 1.** Summary of current and investigational ALS drug treatments including their mechanisms, indications, regulatory status, clinical trial phases, and common side effects.

**Drug/Class**	**Mechanism**	**Indications**	**Regulatory status**	**Trial phase/notes**	**Common side effects**
**Qalsody (Tofersen)**	Antisense oligonucleotide targeting SOD1 mRNA to reduce SOD1 protein	ALS with SOD1 mutation	FDA-approved (2023), EMA pending	Approved based on Phase 3 trial	Injection site reactions, headache, fatigue
**Relyvrio (AMX0035)**	Sodium phenylbutyrate + taurursodiol reducing oxidative stress, mitochondrial dysfunction	ALS (general)	FDA-approved (2022)	Approved based on Phase 2/3 trial	Nausea, diarrhea, abdominal pain
**Radicava (Edaravone)**	Free radical scavenger; reduces oxidative stress	ALS (general)	FDA-approved (2017), EMA approved	Approved Phase 3 trials	Bruising, gait disturbance, headache
**Riluzole (Rilutek, Tiglutik, Exservan)**	Inhibits glutamate release to slow neurodegeneration	ALS (general)	FDA-approved (1995), EMA approved	Long-term approval	Elevated liver enzymes, nausea, fatigue
**Nuedexta (Dextromethorphan/Quinidine)**	NMDA receptor antagonist & sigma-1 receptor agonist; regulates emotional expression	Pseudobulbar affect (ALS and others)	FDA-approved (2010)	Approved for PBA symptoms	Dizziness, diarrhea, QT prolongation risk
**PrimeC (Ciprofloxacin + Celecoxib)**	Targets inflammation, iron accumulation, impaired RNA regulation	ALS (under investigation)	Investigational (no approval yet)	Phase 3 trials ongoing	GI upset, photosensitivity (celecoxib)
**Mexiletine**	Class 1B antiarrhythmic; sodium channel blocker	ALS-related muscle cramps	Off-label use; FDA-approved as antiarrhythmic	Used off-label in ALS for cramps	Nausea, dizziness, cardiac arrhythmia risk
**MSC-NTF Cells**	Neurotrophic factor-secreting mesenchymal stromal cells	ALS (investigational cell therapy)	Investigational	Phase 2/3 trials ongoing	Injection site pain, immune reactions

## 8. Conclusions

ALS remains a devastating condition with limited treatment options. However, ongoing research into its underlying pathophysiology offers hope for future therapeutic developments. In particular, ultra-high field imaging studies may advance our knowledge on this disease by visualizing microstructural changes within the motor cortex and corticospinal tracts that are invisible on conventional 3T scanners potentially allowing us to detect early signs of upper motor neuron degeneration [73]–[75]. A better understanding of genetic factors, as well as early diagnostic methods, could lead to more personalized approaches to managing ALS, thus improving the patient's survival and quality of life.

## Use of AI tools declaration

The authors declare they have not used Artificial Intelligence (AI) tools in the creation of this article.
